# Supplementary material for: Development of Cellular Models to Study Efficiency and Safety of Gene Edition by Homologous Directed Recombination Using the CRISPR/Cas9 System
Source: Cells. 2020 Jun 18;9(6):1492. doi: 10.3390/cells9061492 (PMC7349026; doi:10.3390/cells9061492)
Supplement: Supplementary file 1 [file cells-09-01492-s001.pdf]

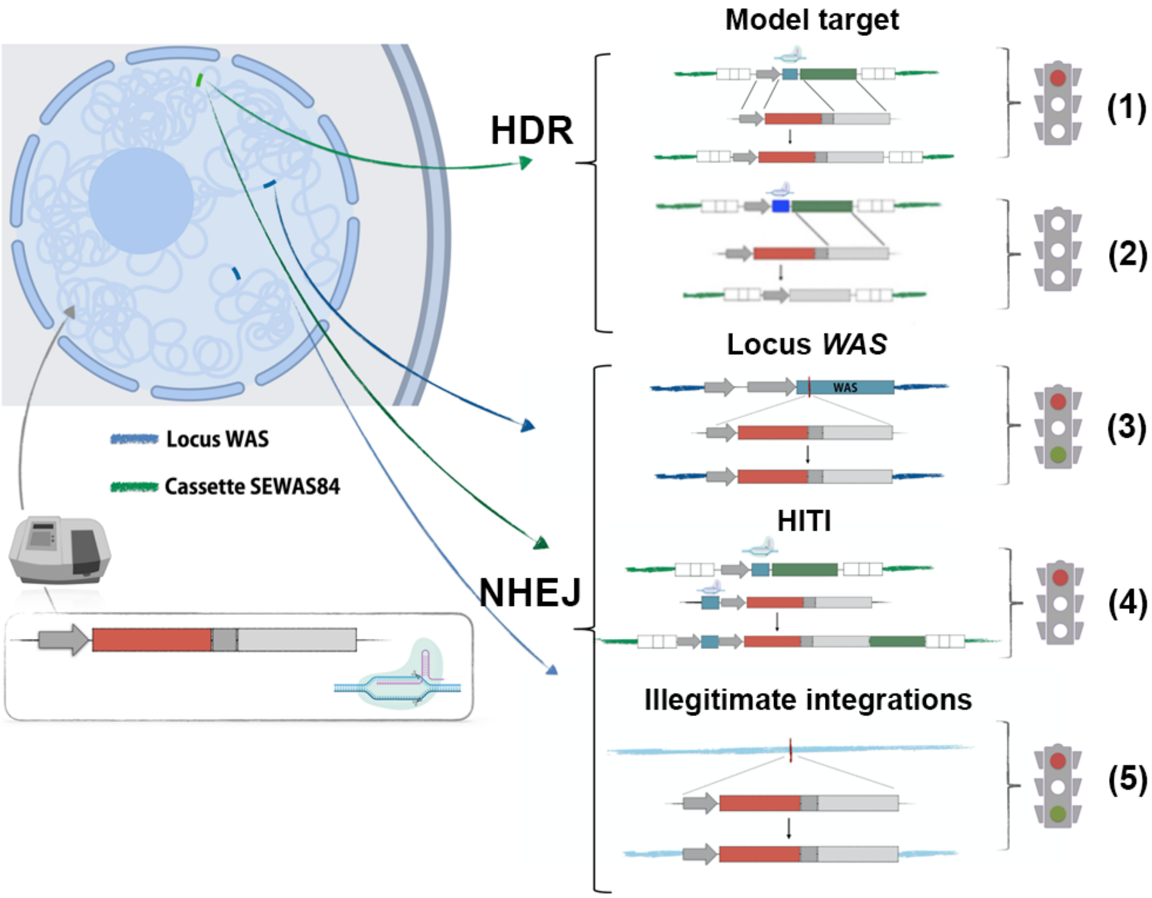

Figure S1. Scheme showing the different possibilities of DNA donor insertions at the K562 SEWAS84 cellular reporter model (eGFP-OFF/dsRED-ON model). Left: nucleus of K562 SEWAS84 cells, with 1 integration of the SEWAS84 cassette (represented in green), and two copies of the endogenous *WAS* locus (represented in blue). Right: The five donor integration possibilities that may occur, 1 and 2 by HDR; 3,4 and 5 by NHEJ: 1) Donor integration by HDR into the target (SEWAS84 cassette), which would mean eGFP-OFF / dsRED-ON. 2) Donor integration by HDR into the target (cassette SEWAS84), but using only the mutated eGFP arm (eGFP*), which would mean eGFP-OFF / dsRED-OFF. 3) Donor integration into the endogenous *WAS* locus, where the original target of the CRISPR/Cas9 gI1 system is located and that will leads to eGFP-ON / dsRED-ON. 4) Full donor integration into the targeted locus through HITI also rendered eGFP-OFF / dsRED-ON. 5) Insertion into other illegitimate sites in the genome as a consequence of CRISPR/Cas off-targets, DSBs generated by physiological conditions or mycrohomology regions. All these situations will also lead to eGFP-ON / dsRED-ON cells.

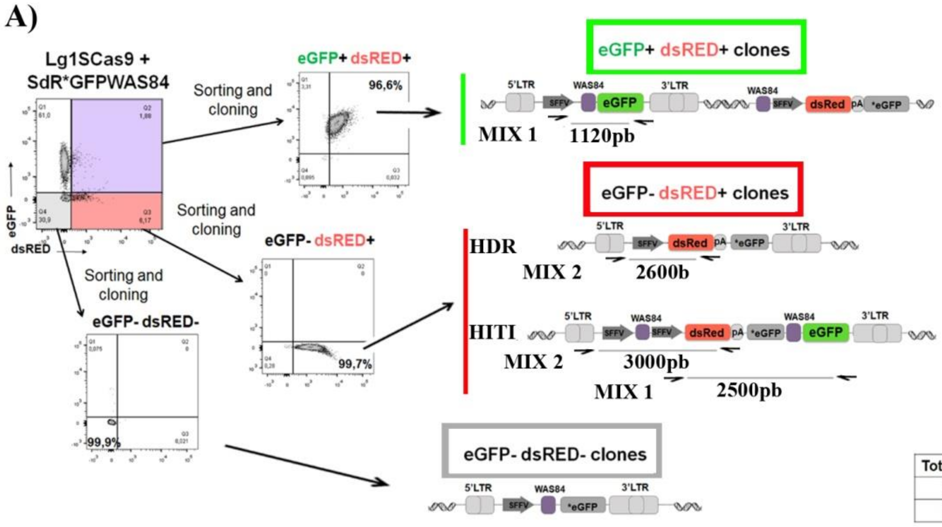

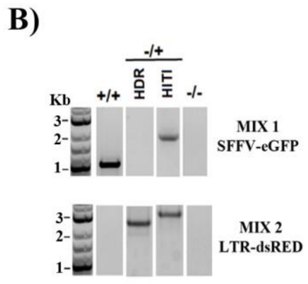

C)

	Clones			
	+/+	-/+		-/-
		HDR	HITI	
Total clones	48	33		26
MIX 1	48	12	21	26
MIX 2	48	12	7	24

Figure S2. Analysis of on-target and off-target donor integration by PCR analysis of clones. A) Left: K562_SEWAS84 cell nucleofected with Lg1SCas9 and SdR*GFPWAS84 donor were expanded over 40 days, sorted and clones to obtain the different emergent subpopulations : GFP+dsdRED+, GFP-dsRED+ and GFP-dsRED- cells. Representative cytometry plots showing eGFP (FITC-A) and dsRED (PE-A) expression of bulk population and clones obtained from each sorted population are shown. The different clones were analyzed by PCR, using the primers depicted at the right each of plot. MIX 1: SFFVfw: AAGAACAGATGGTCCCCAGA and eGFPrev: CGTCCATGCCGAGAGTGA and MIX 2 ΔU3fw: GATCTGCTTTTTGCTTGTACT and dsREDrev: CCTTGTAGATGAAGCAGCCG). Using MIX1 primers, in GFP+dsRED+ clones (off-targets), we expected a 1120bp band corresponding to the original configuration of the target site, since the donor must be integrated in a illegitimate site in order to not interfere with eGFP expression. When MIX1 primers are used in GFP-dsRED+ clones (on target), the expected band will be of 2500pb if HITI occur and no band if HDR is the mechanism (since the eGFP primers only detect the coding cDNA and not the mutated one present in the DNA donor). MIX2 primers are designed to confirm whether the on target integration was due to HDR (2600bp band) or HITI (3000bp band). eGFP-dsRED- clones should not show any band if eGFP has been mutated due to HDR with the 5′ HR as shown in figure S1. B) Example of the analysis performed in the different clones. Each clone was analyzed by PCR using MIX1 (top) and MIX2 (bottom) in order to determine in-target and out-target integrations of the donor DNA. +/+ (eGFP+dsRED+), -/+ (eGFP-dsRED+) and -/- (eGFP-dsRED-). C) Table showing the total number of clones for each population and the number of clones containing donor integration analyzed using Mix1 or Mix2 primers combinations. For GFP-dsRED+ clones (on target) we differentiated those where HDR occurred versus those where HITI was the mechanism to insert the donor DNA in the target locus.

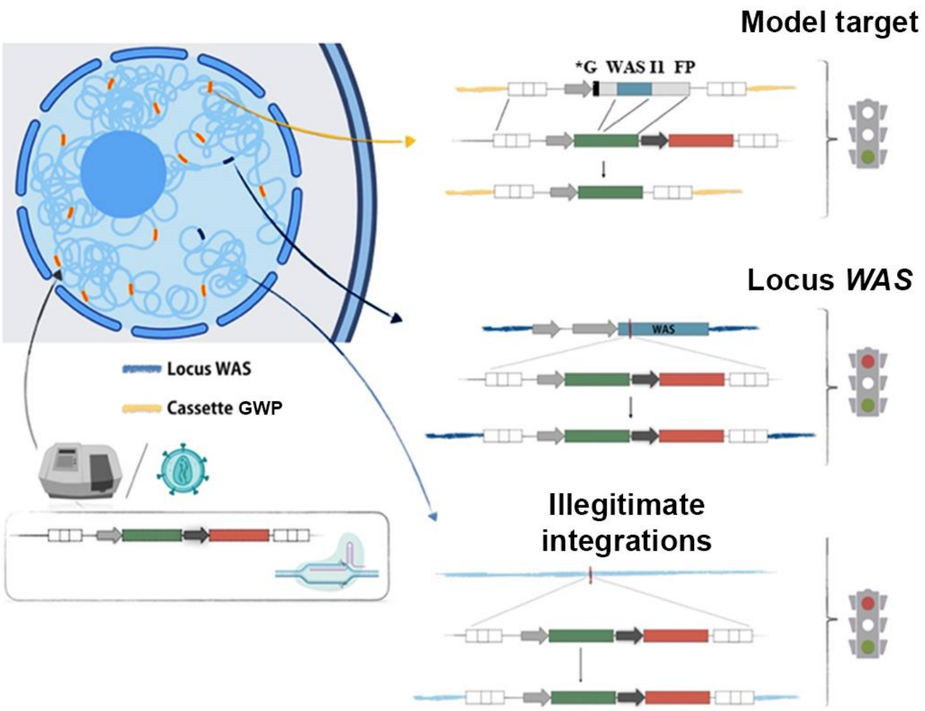

Figure S3. Scheme showing the different possibilities of DNA donor insertions at the 13-K562GWP cellular reporter model (eGFP-ON / dsRED-OFF model). Left: the nucleus of 13-K562GWP cells, with 13 integrations of the SGWP cassette on their genome on average (represented in orange), and two copies of the endogenous *WAS* locus (represented in blue). Right: The three types of donor integrations that may occur. Top: Donor integration by HDR into the target (cassette SGWP), which will generate eGFP+ cells (eGFP-ON / dsRED-OFF). Center: Donor integration into the endogenous WAS locus, where the original target of the CRISPR/Cas9 gI9 system is located. In this case, the cells will appear as GFP+ and dsRED+ as in illegitimate integrations. Bottom: Donor integration into illegitimate sites of the genome again giving rise to double posstive eGFP/dsRED (eGFP-ON / dsRED-OFF).
